# Developmental synapse remodeling in the cerebellum and visual thalamus

**DOI:** 10.12688/f1000research.18903.1

**Published:** 2019-07-25

**Authors:** Masanobu Kano, Takaki Watanabe

**Affiliations:** 1 Department of Neurophysiology, Graduate School of Medicine, The University of Tokyo, Tokyo, 113-0033, Japan; 2 International Research Center for Neurointelligence (WPI-IRCN), The University of Tokyo Institutes for Advanced Study (UTIAS), The University of Tokyo, Tokyo, 113-0033, Japan

**Keywords:** cerebellum, climbing fiber, Purkinje cell, dorsal lateral geniculate nucleus, retinal ganglion cell, development, synapse remodeling

## Abstract

Functional neural circuits of mature animals are shaped during postnatal development by eliminating early-formed redundant synapses and strengthening of necessary connections. In the nervous system of newborn animals, redundant synapses are only transient features of the circuit. During subsequent postnatal development, some synapses are strengthened whereas other redundant connections are weakened and eventually eliminated. In this review, we introduce recent studies on the mechanisms of developmental remodeling of climbing fiber–to–Purkinje cell synapses in the cerebellum and synapses from the retina to neurons in the dorsal lateral geniculate nucleus of the visual thalamus (retinogeniculate synapses). These are the two representative models of developmental synapse remodeling in the brain and they share basic principles, including dependency on neural activity. However, recent studies have disclosed that, in several respects, the two models use different molecules and strategies to establish mature synaptic connectivity. We describe similarities and differences between the two models and discuss remaining issues to be tackled in the future in order to understand the general schemes of developmental synapse remodeling.

## Introduction

In the developing nervous system of neonatal animals, redundant synaptic connections are formed and are present only transiently. Subsequently, some synapses are strengthened whereas other redundant connections are weakened and eventually eliminated ^1– 5^. Such synapse remodeling is widely thought to be essential for shaping functionally mature neural circuits during postnatal development ^4, 5^. Developmental synapse remodeling has been studied in several regions of the brain ^6, 7^ in addition to the neuromuscular junction and autonomic ganglia of the periphery ^8, 9^. Among them, postnatal development of the cerebellar climbing fiber (CF)–to–Purkinje cell (PC) synapse and the retinogeniculate synapse in the dorsal lateral geniculate nucleus (dLGN), the visual thalamus that relays visual information from the retina to the primary visual cortex, are two representative experimental models ^10– 13^. In these two models, postsynaptic neurons receive excitatory synaptic inputs from a limited number of presynaptic axons, and activation of each axon generates relatively large and discernible postsynaptic response. During postnatal development, the majority of the presynaptic axons innervating each neuron are pruned while one or a few axons remain and expand their innervation territories over the postsynaptic neuron. These developmental changes are analogous to those seen in the neuromuscular junction and autonomic ganglia during postnatal development. The CF-to-PC synapse and the retinogeniculate synapse provide simple experimental models of developmental synapse elimination that can be examined quantitatively. Therefore, to gain insight into basic principles of synapse remodeling in the developing brain, we focus on recent advances on the cellular and molecular mechanisms of synapse remodeling in the cerebellum and dLGN.

## Multiple phases of developmental CF-to-PC synapse remodeling in the cerebellum

**Figure 1.**
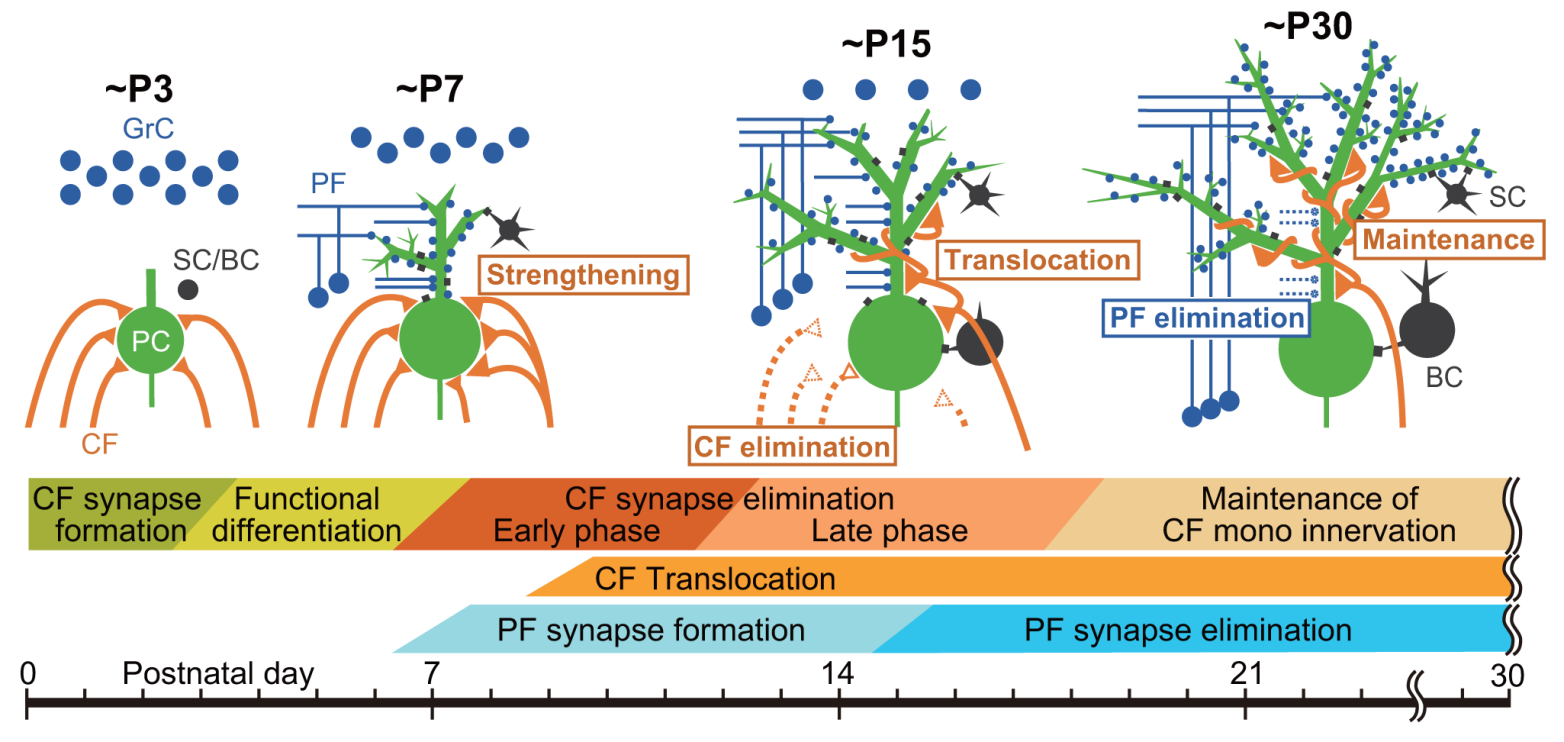
Remodeling of CF and PF synaptic connections onto PCs during postnatal cerebellar development. (Upper panel) Schematics depicting developmental changes in CF and PF synaptic connections to PCs at ~P3, ~P7, ~P15, and ~P30. (Lower panel) Key events related to postnatal development of CF-to-PC and PF-to-PC synapses from birth to ~P30. BC, basket cell; CF, climbing fiber; GrC, granule cell; PC, Purkinje cell; PF, parallel fiber; SC, stellate cell.

In the cerebellum of neonatal rodents, PCs are initially innervated by more than five CFs with similar strengths of synaptic inputs. During the first three postnatal weeks, redundant CFs are eliminated and most PCs become innervated by single CFs ^10, 11, 13^. This developmental process, termed CF synapse elimination, consists of at least four distinct phases in mice ^1, 3, 14^: (1) selective strengthening of a single CF out of multiple CFs innervating each PC from postnatal day 3 (P3) to around P7 (termed “functional differentiation”) ^15^, (2) translocation and expansion of innervation territory of the strongest CF (“winner” CF) to PC dendrites from P9 (termed “dendritic translocation”) ^16^, (3) elimination of somatic synapses of the “winner” CF and those of weaker CFs (“loser” CFs) from P7 to around P11 (termed “early phase of CF elimination”) ^17, 18^, and (4) elimination of the remaining somatic CF synapses from around P12 to P17 in a manner dependent on excitatory synapse formation from parallel fibers (PFs) onto PC dendrites (termed “late phase of CF elimination”) ^18, 19^ ( Figure 1).

Previous studies indicate that neural activity plays crucial roles in CF synapse elimination. In a transgenic mouse line that expressed a chloride channel–YFP fusion specifically in PCs, burst firing of PC was significantly reduced and multiple CF innervation of individual PCs persisted until three months of age ^20^. Andjus *et al*. administered harmaline, which induced synchronous activation of neurons in the inferior olive, to rats from P9 to P12 and altered the normal activity pattern of CFs ^21^. This treatment impaired CF synapse elimination, and PCs remained multiply innervated by CFs up to P87. These results clearly indicate that normal activity levels and firings of postsynaptic PCs and normal activity patterns of presynaptic CFs are both prerequisites for CF synapse elimination.

**Figure 2.**
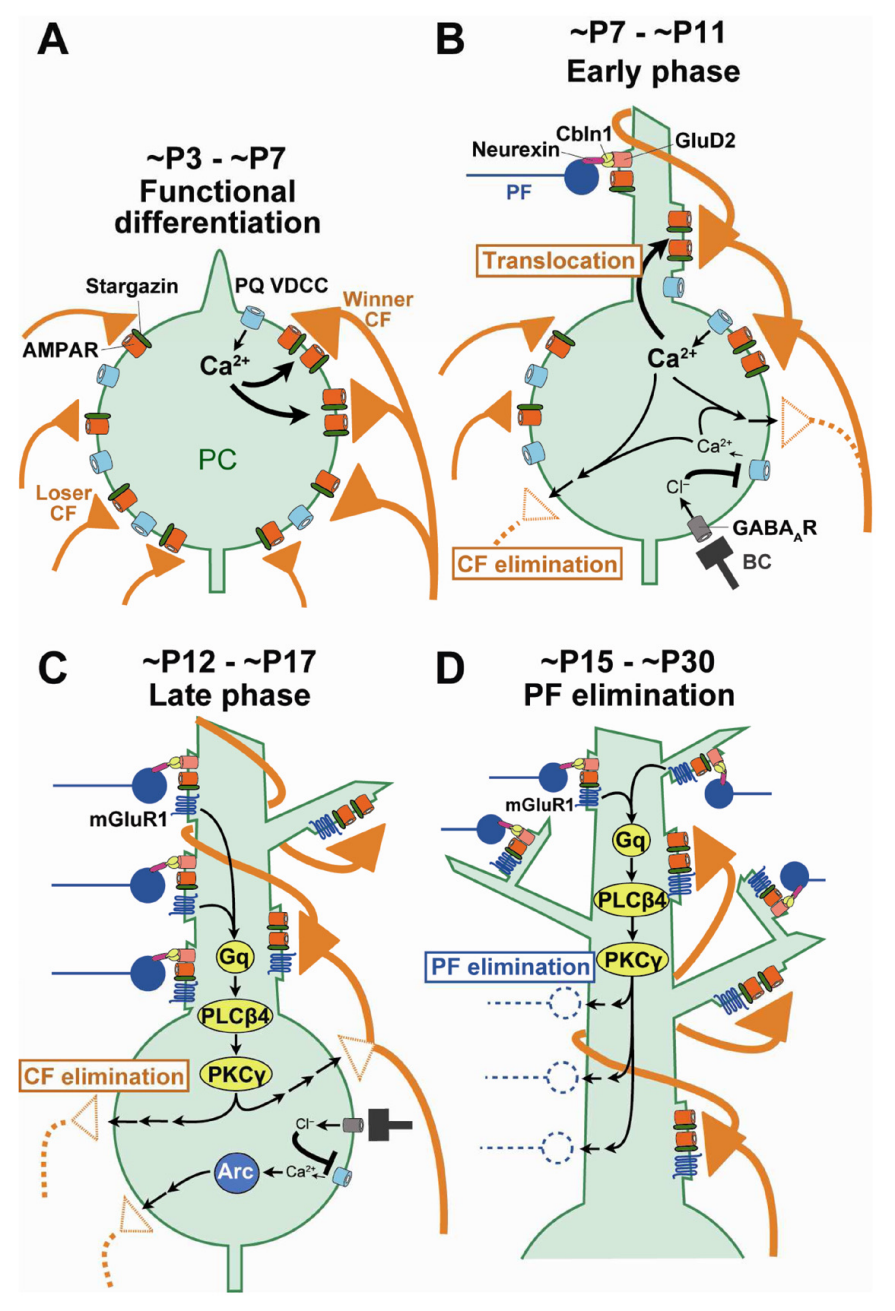
Mechanisms for neural activity-mediated remodeling of CF-to-PC synapses during postnatal cerebellar development. ( **A**) Ca ^2+^ influx through P/Q-VDCC into PCs triggers strengthening of a single CF at ~P3 to ~P7 (functional differentiation). ( **B**) Ca ^2+^ influx through P/Q-VDCC promotes translocation of the single strong CF to the PC dendrite and at the same time eliminates CF synapses from the PC soma at ~P7 to ~P11 (early phase of CF elimination). GABAergic inhibition from BC to PC inhibits Ca ^2+^ influx and thereby regulates elimination of somatic CFs from ~P10. ( **C**) mGluR1 to PKCγ signaling and Arc activated by Ca ^2+^ influx through P/Q-VDCC promotes elimination of somatic CF synapses at ~P12 to ~P17 (late phase of CF elimination). ( **D**) mGluR1 and its downstream signaling in PCs promote elimination of PF synapses from proximal portions of PC dendrites from ~P15 to ~P30 (PF synapse elimination). BC, basket cell; CF, climbing fiber; mGluR1, metabotropic glutamate receptor subtype 1; PC, Purkinje cell; PF, parallel fiber; PKCγ, protein kinase Cγ; VDCC, voltage-dependent Ca ^2+^ channel.

In regard to the molecules that mediate activities of PCs, previous studies have shown that the P/Q-type voltage-dependent Ca ^2+^ channel (VDCC), the major VDCC in PCs, is essential for all four phases of CF synapse remodeling during postnatal cerebellar development ^17, 22, 23^ ( Figure 2). In addition, a study using *in vivo* whole-cell recording from single PCs ^24^ and a recent report of two-photon calcium imaging of PC population activities ^25^ strongly support that P/Q-VDCC is crucial for strengthening of single “winner” CF inputs ( Figure 2). On the other hand, the late phase of CF elimination has been shown to require the metabotropic glutamate receptor subtype 1 (mGluR1)–to–protein kinase Cγ (PKCγ) cascade in PCs ^26– 31^, involves activation of the immediate early gene *Arc* ^22, 32^, and is regulated by GABAergic inhibition of the PC soma by basket cells ^33^ ( Figure 2).

A 2016 study by Ichikawa *et al*. ^34^ revealed that, besides the aforementioned four phases of CF synapse remodeling, massive elimination of PF synapses occurs on PC dendrites from around P15 to P30 ( Figure 1). The authors showed that the domain of PC proximal dendrites with mixed CF and PF innervation expanded vigorously from P9 to P15 because of translocation of winner CFs from the soma to growing dendrites and simultaneous expansion of dendritic territories of PF innervation. Then, from around P15, PF synapses were massively eliminated from the dendritic domain of mixed CF and PF innervation. At P30, CF and PF innervation territories of PC dendrites became segregated such that a single winner CF monopolizes the proximal dendrites and about 100,000 PFs innervate the distal dendrites of each PC. Importantly, PF synapse elimination from P15 to P30 did not occur in mGluR1 or PKCγ knockout mice. Thus, mGluR1-to-PKCγ signaling in PCs is essential for establishing CF mono-innervation of PCs by eliminating redundant CF synapses from the soma and segregating CF and PF innervation territories on PC dendrites by eliminating PF synapses from the proximal dendrites of PCs ^34^ ( Figure 2).

## Retrograde and anterograde signaling molecules for CF-to-PC synapse remodeling

Uesaka *et al*. profiled genes that are expressed in PCs during the period of CF-to-PC remodeling, and they focused on genes that encode secreted or membrane-associated molecules which may function as retrograde signaling molecules from PC to CF ^35^. These candidate genes were screened by performing RNA interference (RNAi) knockdown in PCs ^35^. The authors injected lentivirus carrying microRNA against the candidate gene together with enhanced green fluorescent protein (EGFP) cDNA in the cerebellum of neonatal mice and performed PC-specific knockdown of the candidate gene *in vivo*. Then acute cerebellar slices were prepared at various postnatal days and CF innervation was examined by recording CF-mediated excitatory postsynaptic currents (EPSCs) from PCs with knockdown of the candidate gene and from control PCs in the same slices.

**Figure 3.**
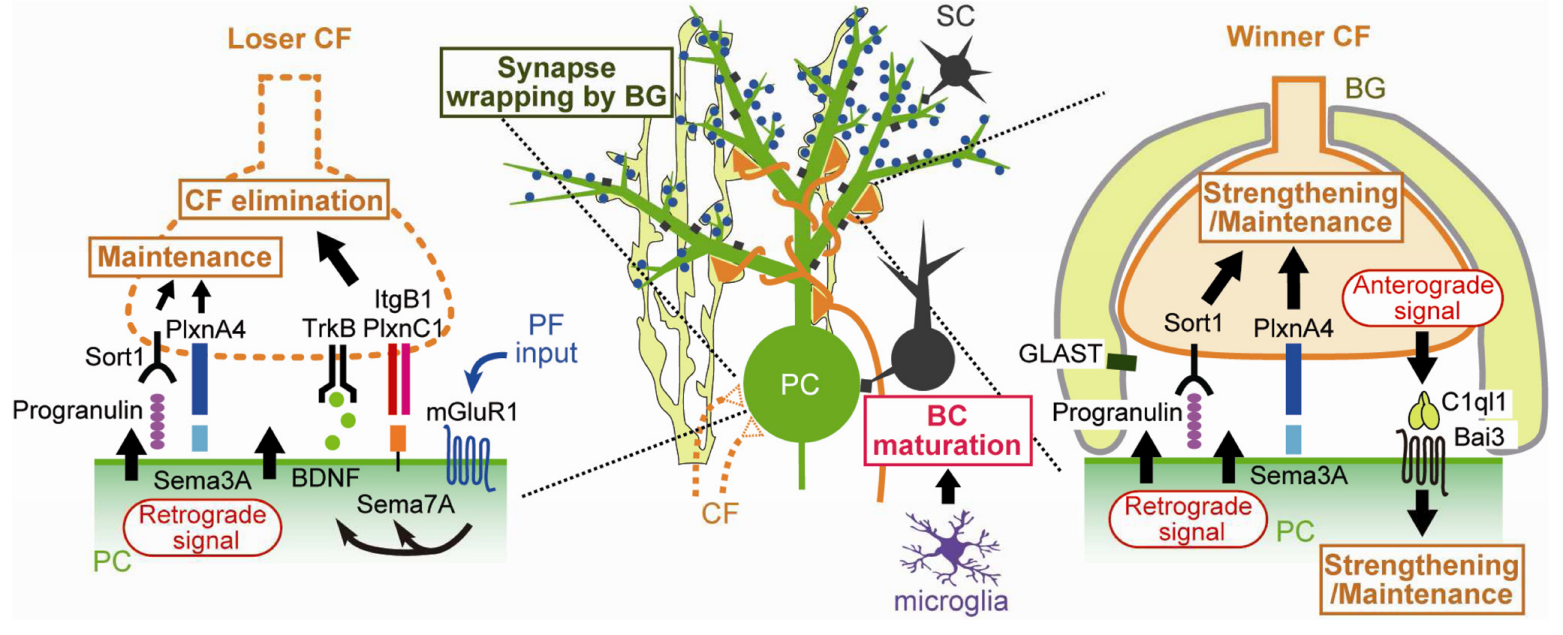
Molecular and cellular mechanisms for transcellular interaction underlying elimination of loser CFs and strengthening/maintenance of winner CFs. Bai3, brain-specific angiogenesis inhibitor 3; BC, basket cell; BDNF, brain-derived neurotrophic factor; BG, Bergmann glia; CF, climbing fiber; GLAST, L-glutamate/L-aspartate transporter; ItgB1, integrin B1; mGluR1, metabotropic glutamate receptor subtype 1; PC, Purkinje cell; PF, parallel fiber; PlxnA4, Plexin A4; PlxnC1, Plexin C1; Sema3A, Semaphorin 3A; Sema7A, Semaphorin 7A; Sort1, Sortilin 1; TrkB, tropomyosin receptor kinase B.

Uesaka *et al*. found that PC-specific knockdown of Sema7A, a membrane-anchored semaphorin, specifically impaired the late phase of CF elimination ^35^. Furthermore, double knockdown of Sema7A and mGluR1 impaired CF synapse elimination to the same extent as single knockdown of mGluR1. By contrast, the effect of Sema7A knockdown and that of either P/Q-VDCC or the glutamate receptor GluD2 knockdown were additive ^35^. These results suggest that Sema7A facilitates the late phase of CF elimination downstream of mGluR1 signaling ( Figure 3). Next, to identify receptors in CFs to which Sema7A acts, Uesaka *et al*. injected lentivirus carrying microRNA against a candidate gene together with EGFP cDNA into the inferior olive, the brainstem nuclei from which CFs originate, of neonatal mice ^35^. Then the authors prepared cerebellar slices at various postnatal ages and examined CF innervation of PCs surrounded by EGFP-positive CFs with knockdown of a candidate gene. Plexin C1 (PlxnC1) and integrin B1 (ItgB1) in CFs were found to function as receptors for Sema7A derived from PCs ( Figure 3). Moreover, focal adhesion kinase (FAK) was found to mediate ItgB1 signaling in CFs ^35, 36^.

Previous studies showed that mice deficient in TrkB, a high-affinity receptor for brain-derived neurotrophic factor (BDNF), were impaired in CF synapse elimination ^37, 38^. Choo *et al*. therefore tested the possibility that BDNF derived from PCs mediates a retrograde signal for CF synapse elimination ^39^ ( Figure 3). They generated PC-specific BDNF knockout mice and also performed lentivirus-mediated BDNF knockdown in PCs of wild-type mice. PC-specific deletion of BDNF was found to impair the late phase of CF elimination. Knockdown of mGluR1 in PCs of PC-specific BDNF knockout mice had no additive effect, whereas knockdown of P/Q-VDCC or GluD2 caused additive impairment of CF synapse elimination in PC-specific BDNF knockout mice. Furthermore, knockdown of TrkB in CFs impaired CF synapse elimination, but the additive effect was not observed in PC-specific BDNF knockout mice. These results suggest that BDNF is released from PCs downstream of mGluR1, retrogradely acts on TrkB in CFs, and facilitates CF synapse elimination during the late phase ^39^ ( Figure 3). To check whether BDNF-to-TrkB signaling interacts with Sema7A-to-PlxnC1/ItgB1 signaling, the effect of Sema7A knockdown in PCs of PC-specific BDNF knockout mice was examined. The effect of Sema7A knockdown was occluded in PCs lacking BDNF, suggesting that the two retrograde signaling pathways converge presumably within CFs ^39^ ( Figure 3).

Besides molecules that facilitate CF synapse elimination, a new category of molecules that strengthen CFs and counteract CF synapse elimination has been identified ( Figure 3). PC-specific knockdown of Sema3A, a secreted semaphorin, accelerated CF synapse elimination from P8 to P18 ^35^. Knockdown of Plexin A4 (PlxnA4), a putative receptor for Sema3A, in CFs also accelerated CF synapse elimination, which was occluded in PCs with Sema3A knockdown ^35, 36^. These results suggest that Sema3A derived from PCs maintains/strengthens CF-to-PC synapses by acting retrogradely via PlxnA4 ^35, 36^ ( Figure 3).

Recently, another set of molecules with a function similar to that of Sema3A and PlxnA4 was identified. Progranulin is a multi-functional growth factor and is known to be implicated in the pathogenesis of certain forms of frontotemporal dementia ^40, 41^. PC-specific deletion of progranulin resulted in acceleration of CF synapse elimination from P11 to P16 ^42^. Knockdown of a putative progranulin receptor, Sort1, in CFs caused acceleration of CF synapse elimination, which was occluded in progranulin-deleted PCs ^42^. The effect of progranulin deletion and that of Sema3A deletion were additive in PCs, suggesting that progranulin and Sema3A maintain/strengthen CF synapses through independent pathways ^42^ ( Figure 3).

Kakegawa *et al*. demonstrated that, in contrast to Sema3A and progranulin, C1ql1, a member of the C1q family protein that is specifically expressed in CFs, strengthened and maintained a single winner CF by anterogradely acting on PCs ^43^ ( Figure 3). This effect was found to be mediated by brain-specific angiogenesis inhibitor 3 (Bai3) in PCs ^43^. Importantly, C1ql1-to-Bai3 anterograde signaling facilitates the elimination of weaker CFs after P9, whereas the same signaling strengthens/maintains the strongest CF and facilitates dendritic translocation of the winner CF ^43^. Thus, the effects of C1ql1-to-Bai3 anterograde signaling are different from those of Sema3A ^35^ and progranulin ^42^ that strengthen/maintain both strong and weaker CFs and therefore counteract the elimination of redundant CFs ( Figure 3).

## Roles of microglia and astrocytes in developmental CF synapse remodeling

In retinogeniculate synapses in the developing dLGN, microglia, which are resident immune cells in the brain, and astrocytes are known to play crucial roles in synapse remodeling by actively removing redundant synapses, which will be discussed later. In contrast, much less was known about the roles of microglia and astrocytes in CF synapse remodeling in the developing cerebellum. Recently, Nakayama *et al*. demonstrated that microglia facilitate CF synapse elimination from P10 ^44^, which largely corresponds to the late phase of CF elimination. Interestingly, the role of microglia in CF synapse remodeling is not phagocytosis of redundant CF synapses but facilitation of inhibitory synapse maturation onto PCs ^44^ ( Figure 3). Nakayama *et al*. found that CF synapse elimination after P10 was impaired in mice whose microglia were ablated by microglia-selective deletion of colony-stimulating factor 1 receptor (Csf1r), a molecule critical for differentiation and survival of microglia in the brain. However, in wild-type mice, the authors found no evidence for engulfment of CFs in microglia. In conditional *Csf1r* knockout mice, GABAergic inhibitory synaptic transmission was impaired. Importantly, enhancing GABA _A_ receptor sensitivity by daily intraperitoneal administration of diazepam from P9 to P12 restored CF synapse elimination. Because maturation of GABAergic inhibition to PCs is known to be required for CF synapse elimination, Nakayama *et al*. conclude that microglia promote maturation of GABAergic inhibition and thereby facilitate CF synapse elimination ^44^ ( Figure 3).

A recent study also shed light on the role of Bergmann glia, specialized astrocytes in the cerebellar cortex, in developmental CF synapse remodeling ^45^. Bergmann glia thoroughly enwrap PCs by extensive lamellate glial processes ^46^ that strongly express the L-glutamate/L-aspartate transporter GLAST ^47, 48^. A previous study demonstrated that PCs of GLAST knockout mice had persistent multiple CF innervation in adulthood ^49^. However, a later study claimed that apparent multiple CF innervation of PCs in GLAST knockout mice resulted mostly from glutamate spillover from CFs innervating neighboring PCs ^50^. A recent detailed study by Miyazaki *et al*. presented clear morphological evidence for multiple CF innervation of PCs in GLAST knockout mice ^45^. The authors found that Bergmann glial processes were retracted from PC dendrites and synapses in GLAST knockout mice. Furthermore, the main ascending CF branch was weakened, whereas the transverse CF branch, which is normally thin and devoid of synaptic terminals, became thick and contained synaptic terminals. Both types of CF branches frequently formed aberrant synaptic connection onto the proximal and distal dendrites of neighboring PCs, which resulted in massive multiple CF innervation in individual PCs. A previous study reported similar phenotypes in mice with virus-mediated expression of the AMPA receptor GluA2 subunit in Bergmann glia ^51^. AMPA receptors in normal Bergmann glia lack GluA2 and are highly permeable to calcium. Although virus-mediated overexpression of GluA2 to Bergmann glia is non-physiological, Bergmann glia with GluA2-containing AMPA receptors were calcium-impermeable, their processes were retracted, and PCs had multiple CF innervation ^51^. These results indicate that the extension of Bergmann glial processes and their complete enwrapping of PCs are prerequisites for establishing CF mono-innervation of PCs by preventing aberrant innervation from CFs innervating neighboring PCs ( Figure 3).

## Multiple phases of developmental retinogeniculate synapse remodeling in the dLGN

**Figure 4.**
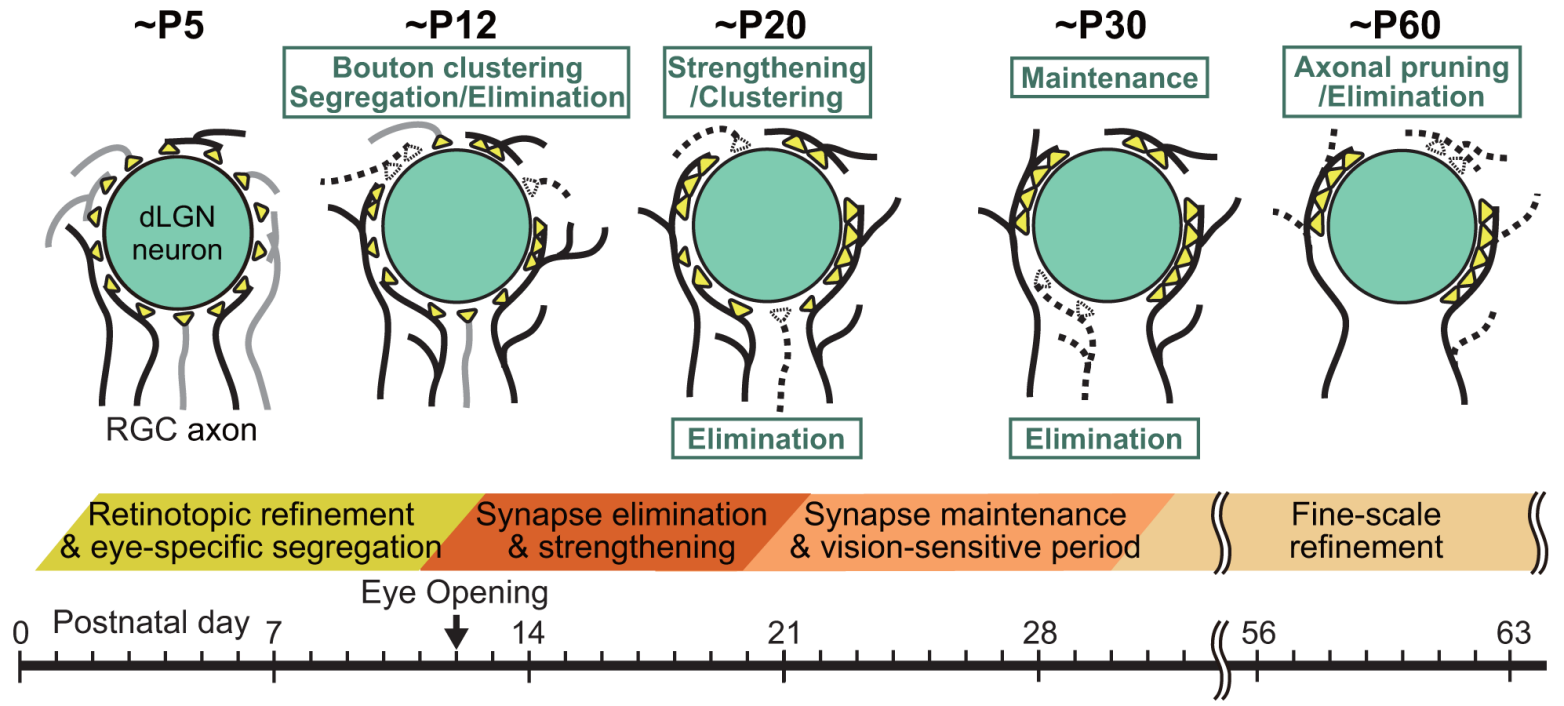
Remodeling of retinogeniculate synapses during postnatal development of the dorsal lateral geniculate nucleus (dLGN). (Upper panel) Schematics depicting developmental changes in retinogeniculate synaptic connections to PCs at ~P5, ~P12, ~P20, ~P30, and ~P60. (Lower panel) Key events related to remodeling of retinogeniculate synaptic connections during postnatal development of the dLGN. RGC, retinal ganglion cell.

In the dLGN, mature retinogeniculate synapses formed by retinal ganglion cell (RGC) axons onto thalamo-cortical (TC) relay neurons are established through at least three distinct phases of postnatal development ^12, 52, 53^ ( Figure 4). First, RGC axons projecting to the dLGN are segregated into eye-specific projection zones by about P10 ^12, 54^ before eye opening at around P12. Each TC neuron receives exuberant weak retinogeniculate synapses at this stage. During the second phase, the average number of RGC axons innervating each TC neuron decreases drastically, whereas a subset of RGC axons per TC neuron become stronger by around P20 ^55, 56^. The first and second phases depend critically on retinal spontaneous activity but not on visual experience ^53^. During the third phase, which spans from about P20 to P30, retinogeniculate synapses are maintained in a visual experience–dependent manner ( Figure 4). Dark rearing of mice from P20 for 1 week (late dark rearing) causes about a twofold increase in the number and about 50% weakening of retinogeniculate afferents to TC neurons ^53, 57^.

**Figure 5.**
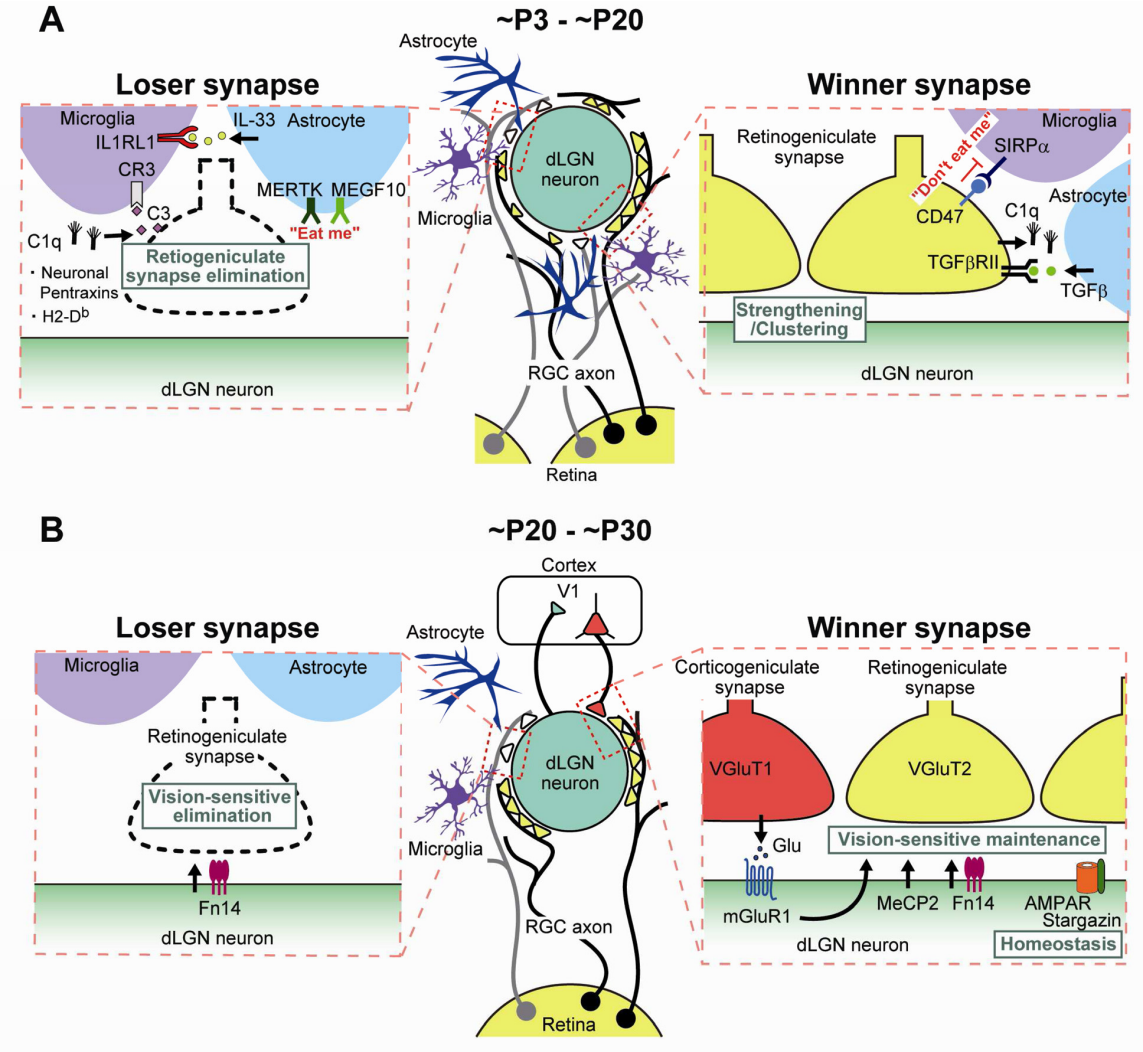
Molecular and cellular mechanisms underlying developmental remodeling of retinogeniculate synapses. Molecular and cellular mechanisms underlying retinogeniculate synapse remodeling ( **A**) from ~P3 to ~P20 (eye-specific segregation and synapse elimination) and ( **B**) from ~P20 to ~P30 (vision-dependent synapse maintenance). dLGN, dorsal lateral geniculate nucleus; IL, interleukin; MeCP2, methyl-CpG-binding protein 2; mGluR1, metabotropic glutamate receptor subtype 1; RGC, retinal ganglion cell; SIRPα, signal regulatory protein alpha; TGF-β, transforming growth factor-beta; V1, primary visual cortex.

Hong *et al*. ^58^ demonstrated that, unlike developmental synapse elimination in the peripheral nervous system ^8, 9^, axon retraction or pruning did not occur during the second and third phases of retinogeniculate synapse remodeling. The authors found that, instead, changes in the size and distribution of presynaptic terminal boutons underlay the functional remodeling of retinogeniculate circuits ^58^ ( Figure 4 and Figure 5). Presynaptic boutons became larger and clustered from around P10 to P20 and underwent dynamic spatial redistribution from around P20 to P30 in response to visual experience ^58^. Furthermore, Hong *et al*. found a fourth phase of retinogeniculate circuit development that follows the experience-dependent refinement phase ^58^. During the fourth phase, significant axon pruning occurred without significant changes in bouton clustering and single fiber strength of retinogeniculate EPSC ^58^ ( Figure 4).

How developmental refinement of retinogeniculate synapse contributes to the maturation of functional properties of visual responses in the dLGN was unknown. Tschetter *et al*. found that spatial receptive fields at the time of eye opening were more than twice as large as in adulthood and decreased in size during the second and third phases of retinogeniculate synapse refinement ^59^. In a slice preparation, excitatory retinogeniculate input decreased and feedforward inhibition increased in TC relay neurons during the period of spatial receptive field refinement ^59^. These results suggest that retinogeniculate synapse remodeling and the resultant change in excitatory/inhibitory balance may underlie the spatial receptive field refinement.

## Roles of the immune system-related molecules in developmental retinogeniculate synapse remodeling

Several molecules related to the immune system have been shown to be involved in eye-specific segregation and subsequent synapse elimination/strengthening of RGC projections in the dLGN ( Figure 5). Huh *et al*. reported that mice deficient in class I major histocompatibility complex (MHC) signaling exhibited incomplete eye-specific segregation ^60^. A later study, by Lee *et al*., demonstrated that mice deficient in the class I MHC molecules H2-K ^b^ and H2-D ^b^ (K ^b^D ^b−/−^) were impaired in eye-specific segregation and synapse elimination/strengthening that were dependent on spontaneous activity of the retina ^61^. The authors also showed that, in dLGN slices from K ^b^D ^b−/−^ mice, long-term depression (LTD) was deficient but long-term potentiation was intact at retinogeniculate synapses ^61^. The impaired synapse refinement and the deficient LTD were both rescued by genetic expression of just H2-D ^b^ in neurons of K ^b^D ^b−/−^ mice ^61^, suggesting that H2-D ^b^ in neurons is necessary and sufficient for structural synapse refinement and functional synaptic plasticity in the dLGN ( Figure 5).

Neuronal pentraxins (NPs), which are homologous to C-reactive and acute-phase proteins in the immune system, were reported to mediate eye-specific segregation of RGC projections ^62^. In mice deficient in both neuronal pentraxin 1 and 2 (NP1 and NP2), eye-specific segregation (examined at P10) was impaired despite relatively normal retinal waves ^62^. Delayed functional maturation of glutamatergic synapses was observed in cultured RGCs ^62^. These results suggest that NPs are required for functional synapse maturation in RGCs and morphological refinement of retinogeniculate synaptic connections.

Another class of immune molecules involved in retinogeniculate synapse refinement is C1q and the downstream molecules of the classic complement cascade, which is part of the innate immune system. Stevens *et al*. found that C1q was expressed in RGCs in response to astrocytes and was localized to synapses during early postnatal development with its peak at around P5 ^63^. The authors demonstrated that eye-specific segregation of RGC projections and subsequent synapse elimination/strengthening were impaired in mice deficient in C1q or its downstream protein, complement component 3 (C3). These results suggest the possibility that redundant synapses are tagged by complement and subsequently eliminated in the developing retinogeniculate system ( Figure 5).

## Roles of microglia and astrocytes in developmental retinogeniculate synapse remodeling

Accumulating evidence suggests that microglia play an important role in synaptic plasticity and remodeling in the healthy brain ^64– 68^. These studies suggest that microglia may phagocytose dendritic spines to shape functionally mature neural circuits. The first experimental evidence for the role of microglia in retinogeniculate synapse remodeling was provided by Schafer *et al*. ^69^. They demonstrated that microglia engulfed presynaptic retinal inputs during peak retinogeniculate synapse pruning at P5. The engulfment by microglia was dependent on neural activity as blockade of retinal activity by injecting tetrodotoxin into the eye reduced the extent of engulfment ^69^. Furthermore, the microglial engulfment of presynaptic inputs required a phagocytic receptor, complement receptor 3 (CR3), on the surface of microglia, and its ligand, C3, localized to regions with enriched synapses ^69^. Disruption of microglia-specific CR3/C3 signaling impaired eye-specific segregation and caused the persistent presence of redundant retinogeniculate synapses ^69^. Thus, C1q and C3 are considered to function as an “eat me” signal to eliminate unwanted weak retinogeniculate synapses by promoting microglial engulfment through CR3 ( Figure 5).

A recent study identified “don’t eat me” signals that appear to protect more active retinogeniculate synapses from microglial phagocytosis ^70^. Lehrman *et al*. demonstrated that CD47-to-SIRPα signaling prevented excess pruning of retinogeniculate synapses through microglial phagocytosis ^70^. CD47 is an immunoglobulin superfamily protein on the cell surface and inhibits phagocytosis by binding to its receptor, SIRPα, on professional phagocytes ^71, 72^. Mice deficient in CD47 or SIRPα showed enhanced microglial engulfment and CD47 knockout mice had a persistently reduced synapse number because of enhanced synapse pruning ^70^. CD47 was preferentially localized to more active synapses, whereas SIRPα was highly expressed by microglia during peak synapse pruning ^70^. Thus, these results suggest that CD47-to-SIRPα signaling is regulated by neuronal activity and specifically protects active retinogeniculate synapses from microglial phagocytosis ( Figure 5).

Astrocytes are also shown to play a key role in phagocytosis and pruning of retinogeniculate synapses during postnatal development ^73^. MEGF10 and MERTK are phagocytic receptors that recognize “eat me” signals on cell debris ^74, 75^. Chung *et al*. demonstrated that both MEGF10 and MERTK were localized in astrocytes and they engulfed synapses of retinogeniculate inputs during peak synapse pruning ^73^ ( Figure 5). Mice deficient in both phagocytotic receptors are impaired in eye-specific segregation and synapse elimination/strengthening in the dLGN ^73^. This astrocyte-mediated synapse pruning was promoted by retinal neural activity. Importantly, the authors quantitatively estimated the relative contribution of astrocytes and microglia to phagocytosis of synapses in the developing dLGN. The amount of engulfed retinogeniculate synaptic debris per imaging field by astrocytes was about 4- to 10-fold larger than that by microglia from P3 to P9, suggesting that the total amount of synapse pruning by astrocytes may exceed that by microglia ^73^.

Besides directly phagocytosing synapses, astrocytes regulate or influence functions of microglia. During peak synapse pruning in the dLGN, astrocytes have been reported to release transforming growth factor-beta (TGF-β) that is sensed by TGF-β receptor II expressed in RGCs ^76^. The astrocyte-derived TGF-β regulated C1q expression in RGCs and thereby controlled the degree of microglial engulfment of RGC inputs ^76^ ( Figure 5). Furthermore, a recent study in the developing spinal cord and somatosensory thalamus showed that the interleukin 1 (IL-1) family cytokine IL-33 derived from astrocytes promoted synapse engulfment by microglia ^77^. Thus, astrocytes appear to control microglial phagocytosis of synapses in the developing brain by releasing TGF-β and IL-33 ( Figure 5).

## Mechanisms for visual experience–dependent maintenance of refined retinogeniculate synapses

Studies in the past several years have disclosed how retinogeniculate synapses that have undergone eye-specific segregation and synapse elimination/strengthening are maintained during the third phase in a visual experience–dependent manner. In mice deficient in methyl-CpG-binding protein 2 (MeCP2) whose mutations underlie the neurodevelopmental disorder Rett syndrome ^78, 79^, initial synapse formation, eye-specific segregation, and synapse elimination/strengthening up to P21 were normal ^80^ ( Figure 5). However, during the experience-dependent maintenance phase, the number of RGC inputs per relay neurons increased, retinal inputs did not strengthen further, and the late dark rearing had no further effect in MeCP2 knockout mice ^80^. Furthermore, specific impairment of the experience-dependent phase of synapse refinement has been reported in mice lacking the AMPA receptor auxiliary subunit stargazin ^81^ ( Figure 5). Visual deprivation increased stargazin expression and phosphorylation, which caused reduced rectification of AMPAR-mediated EPSCs ^81^. Furthermore, stargazin phosphorylation was found to be essential for synaptic scaling ^81^.

Thompson *et al*. reported that feedback inputs from the primary visual cortex (V1) to the dLGN regulated the visual experience–dependent phase of retinogeniculate synapse refinement ^82^. Pharmacological and chemogenetic suppression of cortical feedback activity from the V1 during P20 to P27 increased the number of RGCs innervating each thalamic relay neuron ^82^. Importantly, chemogenetic enhancement of V1 activity from P20 to P27 induced similar synaptic rewiring, suggesting that maintenance of a mature pattern of retinogeniculate connectivity from P20 to P27 requires proper levels or patterns of cortical feedback activity or both ^82^ ( Figure 5).

The mGluR1 is richly expressed in the thalamic nuclei including the dLGN ^83, 84^. Narushima *et al*. demonstrated that, in mGluR1 knockout mice, visual experience–dependent maintenance of retinogeniculate synapses was specifically impaired ^85^. Similar impairment was observed by RNAi-mediated knockdown and pharmacological blockade of mGluR1 in the dLGN ^85^. Late dark rearing had no effect in mGluR1 knockout mice, and the effect of late dark rearing in wild-type mice was rescued by pharmacological activation of mGluR1 in the dLGN ^85^. Quantitative immune-electron microscopic examination revealed that mGluR1 was preferentially expressed at postsynaptic sites of cortico-geniculate synapses ^85^. Therefore, it is legitimate to assume that mGluR1 mediates cortical feedback activity for the maintenance of retinogeniculate synapses ( Figure 5).

Recently, single-cell RNA sequencing was employed to obtain a whole transcriptome database of gene expression induced by visual experience in excitatory and inhibitory neurons in the developing dLGN ^86^. Among hundreds of such genes, the cytokine receptor Fn14 was the most inducible molecule in excitatory neurons. Mice deficient in Fn14 exhibited normal retinogeniculate synapse refinement mediated by spontaneous retinal activity but impaired synapse maintenance during the visual experience–dependent phase ^86^. Fn14-deficient mice have functionally weaker and morphologically smaller retinogeniculate synapses compared with wild-type mice ^86^. Thus, Fn14 appears to be a molecule that links visual experience and synapse refinement in the dLGN ( Figure 5).

## Conclusions

Recent studies have disclosed new molecules and cellular interactions that are involved in developmental synapse remodeling in the cerebellum and dLGN. By reviewing synapse remodeling in the two brain regions, similarities and differences have come to light (as summarized below). In the dLGN, bouton clustering but not axon pruning contributes to synapse remodeling during the three phases until around P30 ^58^. In contrast, it is not clear whether similar bouton clustering occurs and contributes to CF-to-PC synapse remodeling in the developing cerebellum. In both the cerebellum and the dLGN, neural activity is essential for developmental remodeling of CF-to-PC synapses ^17, 20, 22, 24, 32, 87, 88^ and retinogeniculate synapses ^53, 89, 90^, respectively. In the dLGN, spontaneous retinal activity is necessary for the first and second phases until P20, whereas visual inputs are crucial for the third phase ^53^. In contrast, it is not clear whether spontaneous activity or some external drive such as somatosensory input is required for any of the four phases of CF-to-PC synapse remodeling. Nevertheless, normal activity levels and firing patterns of postsynaptic PCs ^20^ and normal activity patterns of presynaptic CFs ^21^ are both required for CF synapse elimination. As for the molecules mediating neural activity, P/Q-VDCC ^17^ and mGluR1 in PCs ^26, 28, 31^ have been identified for CF-to-PC synapse remodeling whereas data for the molecules mediating neural activity for retinogeniculate synapse remodeling are limited. Whereas mGluR1 appears to mediate cortical feedback inputs from V1 to TC relay neurons during the third phase ^85^, molecules involved in spontaneous retinal activity–dependent synapse remodeling during the first and second phases are not clear. In regard to trans-synaptic molecules involved in pruning or strengthening of synaptic connections, several key molecules, including Sema7A ^35^, Sema3A ^35^, BDNF ^39^, progranulin ^42^, and C1ql1 ^43^, have been identified for CF-to-PC synapse remodeling whereas little is known for retinogeniculate synapse remodeling. On the other hand, several immune system–related molecules, including H2-D ^b^ ^61^, NPs ^62^, and C1q, C3, and CR3 ^63, 69^, play important roles in the first and second phases of retinogeniculate synapse remodeling. However, contributions of such immune system–related molecules to CF-to-PC synapse remodeling have not been reported. Furthermore, both microglia and astrocytes directly phagocytose unwanted retinogeniculate synapses ^69, 73^, and molecules controlling the synapse phagocytosis, including C3 and CR3 ^69^, MEGF10 and MERTK ^73^, and CD47 and SIRPα ^70^ as well as TGF-β ^76^ and IL-33 ^77^, have been reported. In contrast, microglia do not engulf CF-to-PC synapses during the postnatal period but promote the development of inhibitory synapses and thereby facilitate CF synapse elimination ^44^.

Future studies should further investigate similarities and differences between the two models of developmental synapse remodeling. The molecules that have been identified as involved in one model should be tested in the other provided that the same or similar molecules are also expressed. Furthermore, the molecules that are required for the two models should be tested in synapses in other brain regions such as the hippocampus and cerebral cortex. Such studies will highlight the uniqueness and commonality of synapse remodeling in different brain regions and may ultimately lead to the uncovering of the common principle of functional neural circuit formation during postnatal development.

## Abbreviations

Bai3, brain-specific angiogenesis inhibitor 3; BDNF, brain-derived neurotrophic factor; C3, complement component 3; CF, climbing fiber; CR3, complement receptor 3; Csf1r, colony-stimulating factor 1 receptor; dLGN, dorsal lateral geniculate nucleus; EGFP, enhanced green fluorescent protein; EPSC, excitatory postsynaptic current; GLAST, L-glutamate/L-aspartate transporter; IL, interleukin; ItgB1, integrin B1; LTD, long-term depression; MeCP2, methyl-CpG-binding protein 2; mGluR1, metabotropic glutamate receptor subtype 1; MHC, major histocompatibility complex; NP, neuronal pentraxin; P, postnatal day; PC, Purkinje cell; PF, parallel fiber; PKCγ, protein kinase Cγ; PlxnA4, Plexin A4; PlxnC1, Plexin C1; RGC, retinal ganglion cell; RNAi, RNA interference; TC, thalamo-cortical; TGF-β, transforming growth factor-beta; VDCC, voltage-dependent Ca ^2+^ channel; V1, primary visual cortex
